# Evaluation of the Correlation between Focal Adhesion Kinase Phosphorylation and Cell Adhesion Force Using “DEP” Technology

**DOI:** 10.3390/s120505951

**Published:** 2012-05-08

**Authors:** Chyung Ay, Chih-Chang Yeh, Min-Chih Hsu, Huaang-Youh Hurng, Philip Chi Lip Kwok, Hsin-I. Chang

**Affiliations:** 1 Department of Biomechatronic Engineering, National Chiayi University, No. 300, University Road, East District, Chiayi 600, Taiwan; E-Mails: cay@mail.ncyu.edu.tw (C.A.); lpslbsleo531@yahoo.com.tw (M.-C.H.); hungyu@mail.ncyu.edu.tw (H.-Y.H.); 2 Department of Orthopaedics, Chiayi Branch, Taichung Veterans General Hospital, No.600, Sec. 2, Shixian Road, West District, Chiayi City 60090, Taiwan; E-Mail: yeh215kimo@yahoo.com.tw; 3 Department of Pharmacology and Pharmacy, Li Ka Shing Faculty of Medicine, The University of Hong Kong, Pokfulam, Hong Kong; E-Mail: pclkwok@hku.hk

**Keywords:** DEP force, focal adhesion kinase, cell adhesion force, collagen, fibronectin

## Abstract

Dielectrophoresis (DEP) is the phenomenon in which a particle, such as a living cell, is polarized and moved by electrical gravity in a non-uniform electric field. In the present study, the DEP force is utilized to act on the cells to induce spatial movement for investigating the correlation between the cell adhesion force and activation level of focal adhesion kinase (FAK). The DEP force produced by the non-uniform electric field was used to measure the cell adhesion force of ECV304 cells, on type 1 collagen (COL1)- and fibronectin (FN)-coated polydimethylsiloxane (PDMS) membranes. For COL1-coating, ECV304 cells revealed weak and variable adhesion force (0.343–0.760 nN) in the first eight hours of incubation. Interestingly, the cell adhesion force of ECV304 at two and five hours of cultivation was significantly high and matched their FAK activation level. In comparison, ECV304 on FN-coated membrane had higher and more stable cell adhesion force (0.577–2.053 nN). FN coating intensified the cell adhesion force of ECV304 with culture time and similar outcome was present on the activation level of FAK. Therefore, this study demonstrated a relationship between cell adhesion force and FAK activation level that was dependant on the choice of the extracellular matrix (ECM) component. Subsequently, two tyrosine kinase inhibitors (AG18 and genistein) and one PI3K inhibitor (LY294002) were applied to study the influence of protein phosphorylation on the cell adhesion force. FAK plays an important role on cell attachment and DEP force measurement is a useful technique for studying cell adhesion.

## 1. Introduction

The cell-extracellular matrix (ECM) adhesion not only plays a important role in cell functions, including morphology, spreading, migration, and differentiation, but is also a key step in the tissue engineering approach for maintenance of tissue structure and cell integration of biomaterials [1,2]. ECM components for cell adhesion, such as type 1 collagen (COL1), laminin (LM), and fibronectin (FN), are primarily mediated by integrin on the cell surface [3]. Integrin-mediated cell-ECM interactions promote the assembly of cytoskeletal and signaling molecule complexes at sites of focal adhesions (FAs). FAs are formed at the cell-ECM contact points, where bundles of actin filaments are anchored to transmembrane receptors of the integrin family through a multi-molecular complex of junctional plaque proteins [4]. These complexes include Src-family members, focal adhesion kinase (FAK), phosphatidylinositol-3-kinase (PI3K), and phospholipase C (PLC)-γ [5]. A number of studies highlight that FAK plays an important role in regulation of cell spreading, migration, and adhesion strength. Inhibition of FAK by over-expression of its non-catalytic COOH-terminal domain resulted in decreased rate of cell spreading and cell migration [6,7]. Furthermore, it has been reported that FAK expression in FAK-null fibroblasts enhanced integrin activation and cell adhesion strength [8]. While the roles of FAK in cell adhesion strength have been established, the contributions of ECM components through FAK to cell adhesion strength remain unclear. One aim of the present study was to investigate the role of FAK on cell adhesion force in human bladder epithelial cells, ECV304, under the stimulus of COL1 and FN.

Several studies have investigated the cell adhesion force by mechanical approaches. In previous studies, cell adhesion has been studied as centrifugal force by centrifugation, tensile force by micropipette manipulation, shear force by parallel flow chamber, and chemical binding force by atomic force microscopy (AFM) [9–12]. Furthermore, Curtis and Spatz applied holographic optical tweezer techniques to study hyaluronan-mediated adhesion processes of chondrocyte cells [13]. Rico *et al.* modified silicon pyramidal AFM cantilever tips to flat-ended cylindrical tips and Shen *et al.* fabricated micro-pullers and nano-pickers from AFM cantilevers for cell adhesion measurement by AFM [14–16]. In the present study, dielectrophoresis (DEP) force was ultilized to induce cellular movement in a non-uniform electric field to investigate cell adhesion. DEP has been used for cell characterization and manipulation for a long time because DEP force can capture and categorize cells through applied AC electrical field gradients [13,17]. For example, Lapizco-Encinas *et al.* utilized DEP across a microchannel system to concentrate and selectively release live and dead *Escherichia coli* [18]. Most studies utilizing DEP employ sophisticated planar DEP microelectrode arrays coupled to microfluidic systems for large-scale separation of thousands of cells [17–19]. Like gel electrophoresis, which moves particles in a uniform, constant field has been widely applied for the separation and analysis of a variety of biological particles such as cells, DNA, and viruses, DEP may provide a new technique in cell adhesion measurement. In our present study, we demonstrated that DEP can be used to investigate the interaction between cells and ECM components and FAK regulates cell adhesion force under the stimulus of COL1 and FN.

## 2. Experimental Section

### 2.1. Materials

Human bladder epithelial cells, ECV304 was obtained from the American Type Culture Collection (ATCC). SYLGARD^®^ 184 silicone elastomer kit was purchased from Dow Corning (Taipei, Taiwan). All culture materials were purchased from Gibco (Grand Island, NY, USA) and all chemicals of reagent grade were obtained from Sigma (St Louis, MO, USA). Polydimethylsiloxane (PDMS) membranes were prepared with SYLGARD^®^ 184 silicone elastomer base and SYLGARD® 184 silicone elastomer curing agent in the ratio of 10 to 1. After the polymer mixture was poured into the mould, the mould was placed in a vacuum chamber for 30 min to remove air bubbles and heated to 100 °C within an hour for PDMS solidification. After 1 min of plasma treatment, 50 μL of type 1 collagen (100 mg/mL, 1% w/v) or fibronectin (100 mg/mL, 1% w/v) were spreaded on PDMS membrane for COL1 or FN coating. Finally, we measured the contact angle of PDMS membranes to ensure that the COL1 or FN coating was formed. This was shown by a reduction in the contact angle from 107.6° to 0°.

### 2.2. Theoretical Background on DEP Force

DEP force is a phenomenon in which a force is exerted on a dielectric particle when it is subjected to a non-uniform electric field. The movement of the particles (cells) depends on the cellular properties, working solution, and the strength of the electrical field. The dielectrophoresis force *F*(x020D7)*_DEP_* acting on a homogeneous dielectric ellipsoidal particle is [20,21]:

(1) $${\vec{F}}_{\text{DEP}}=\frac{3}{2}\nu \varepsilon_{m}\text{Re}\left[K_{n}(\omega )\right]\nabla {\left|E_{\text{rms}}\right|}^{2}$$

where *v* is the particle (cell) volume, *ε_m_* is the permittivity of the suspending medium, ∇|E_rms_|^2^ is gradient of the root mean square value of the electric field squared, and *K_n_*(*ω*) is a frequency dependent factor. Re[*K_n_* (*ω*)] is the real part of *K_n_*(*ω*). The frequency dependent factor is given by:

(2) $$K_{n}(\omega )=\frac{\varepsilon_{p}^{*}-\varepsilon_{m}^{*}}{3(A_{n}(\varepsilon_{p}^{*}-\varepsilon_{m}^{*})+\varepsilon_{m}^{*})}$$

where:

(3) $$A_{n}=\frac{1}{2}r_{1}r_{2}r_{3}\int_{0}^{\infty }\frac{ds}{(s+r_{n}^{2})B}$$

and where $\varepsilon_{m}^{*}$ and $\varepsilon_{p}^{*}$ are the complex permittivities of the suspending medium and particle, respectively. In this case, the cell is akin to a particle and glucose solution is the suspending medium. A general complex permittivity is defined as ε* = *ε -j(σ/ω)* with permittivity *ε* and conductivity *σ*. Where $j=\sqrt{-1}$ and *ω* is the frequency, *A_n_* is depolarising factor for the axis *n*, $B\sqrt{\left(s+r_{1}^{2})+(s+r_{2}^{2})+(s+r_{3}^{2}\right)}$ and *s* is an arbitrary distance for integration, and *r*_1_, *r*_2_, *r*_3_ are the half lengths of the major axes *n* (*n* = 1, 2, 3) of the particle. For a sphere, *r*_1_ = *r*_2_ = *r*_3_ and *A*_1_ = *A*_2_ = *A*_3_ = 1/3, the frequency dependent factor in Equation (2) is:

(4) $$K_{n}(\omega )=\frac{\varepsilon_{p}^{*}-\varepsilon_{m}^{*}}{\varepsilon_{p}^{*}+2\varepsilon_{m}^{*}}$$

where *K*(*ω*) is referred to as the Clausius-Mossotti factor. Definitions of the other terms in Equation (4) are listed below:

ε_0_: dielectric constant in vacuum, 8.854 × 10^−12^ (F/m)
$\varepsilon_{m}^{*}$: dielectric constant for work solution (glucose), 76.5 ε_0_ (F/m)
$\varepsilon_{p}^{*}$: dielectric constant for bioparticl, 60 ε_0_ (F/m)
σ_m_: conductivity for work solution (glucose), 3.53 (μS/cm)
σ_p_ conductivity for bioparticle, 0.5 (S/m)

The direction of the force (positive or negative DEP) is governed by the value of Re[*K*(*ω*)], which can be positive or negative, respectively. If Re[*K*(*ω*)] is positive, particles move to regions of highest field strength (positive dielectrophoresis). In contrast, negative DEP (Re[*K*(*ω*)] < 0) forces cause particles to be repelled from the maximum electric field gradient. In this study the liquid medium is glucose (2% w/v) and Re[*K*(*ω*)] was calculated to be +0.999.

### 2.3. Cell Culture

The human bladder epithelial cells, ECV304, were cultured in Medium 199 supplemented with 10% w/v fetal bovine serum, 2 mM glutamine, 100 U/mL penicillin and 100 μg/mL streptomycin. For cell adhesion measurement and analysis of FAK phosphorylation, ECV304 cells were cultured on 1% w/v COL1- and 1% w/v FN-coated polydimethylsiloxane (PDMS) membranes and the seeding density was 5,000 cells/cm^2^. Due to the poor hydrophilicity of PDMS, the PDMS membranes required oxygen plasma pretreatment to improve surface tension for the coating of COL1 and FN.

### 2.4. Measurement of Cell Adhesion Force

**Figure 1.**
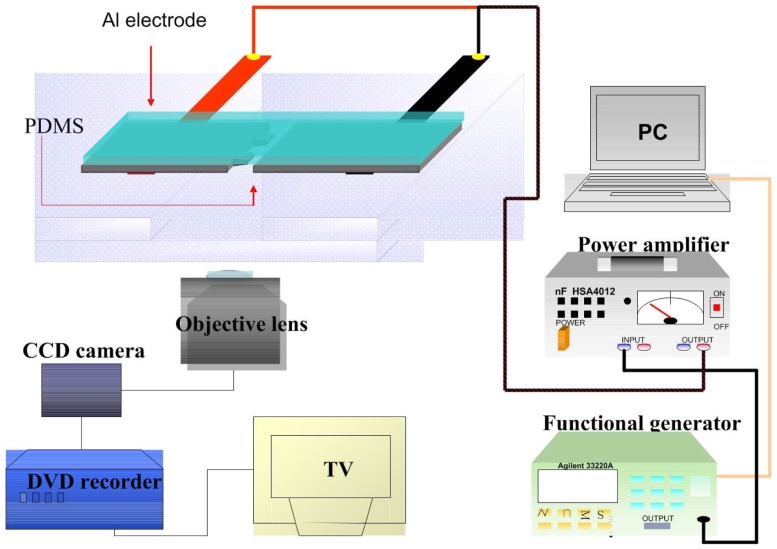
The scheme of dielectrophoresis (DEP) force measurement system for determining the cell adhesion strength of ECV304 on COL1 or FN coated PDMS membrane. The electrophoresis force is produced by the driving voltage of electrodes.

**Figure 2.**
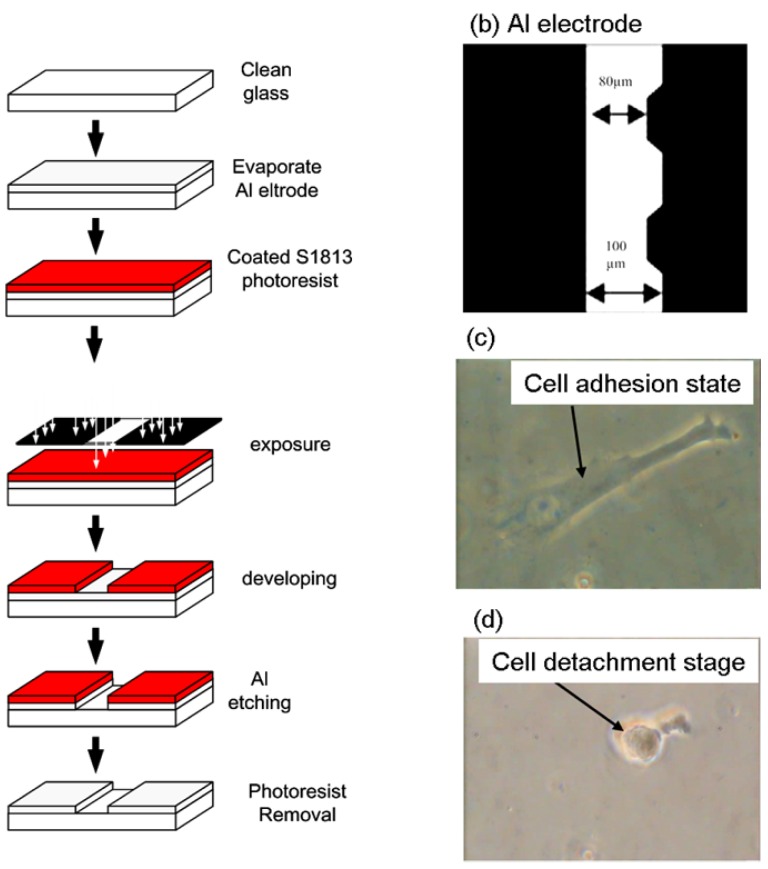
The manufacture scheme (**a**) and structure (**b**) of a single-layer aluminum electrode and cell morphology before (**c**) and after cell detachment (**d**).

Cell adhesion force was measured by dielectrophoresis. Figure 1 shows the experimental setup consisting of an optical microscope (Model CK30, Olympus, Japan), a charge coupled device (CCD) camera (Model E-330, Olympus, Japan), a function generator (Model 33220A, Agilent, USA), a power amplifier (Model HSA4012, NF Corporation, Japan), and aluminum (Al) electrodes. The experiment employed micro-processing technology to etch a glass plate with two electrodes of different widths (80 μm and 100 μm) to produce a non-uniform electric field. To produce the aluminum electrodes, a photo resistor rotary coater was used to create a positive photo resistor S1813 coating (step 1: 500 rpm/5s, step 2: 6,000 rpm/30s) on a glass cover slip, which was subsequently baked in an oven at 115 °C for 25 min. After baking, the cover slip was exposed for 90 s and developed for 40 s, followed by rinsing with deionized (DI) water and aluminum etching with a solution containing 70% phosphoric acid, 3% nitric acid, 14% acetic acid, and 13% water. The photo resistor was finally removed with acetone (Figure 2(a)).

Figure 2(b) shows a microscopic image of the resultant electrode on the cover slip. The Al electrodes were about 300 nm thick, with an 80 μm gap between the electrodes. The cover slip of the fabricated electrode was immersed in 95% alcohol for 1 h and then in DI water for 2 h before starting the experiment. For DEP force generation, an electrical potential was applied to the electrodes by a function generator coupled to a power amplifier to produce a non-uniform electric field.

For measurement of cell adhesion force, a single cell was cultured on a 1% COL1- or FN-coated PDMS membrane (Figure 2(c)). At certain time intervals after cell culture, the PDMS membranes were covered with the electrode plate. The cell adhesion force was determined by the cell's movement away from the seeding position when an external electrical field was applied to cause dielectrophoresis. The external AC electrical field (1 MHz) was set at 0 V at the beginning of the experiment and was increased steadily until the cell moved. During the measurement of cell adhesion, the electrical potential was increased at 1/3 V/s from 0 to 10 V and then changed to 1/10 V/s until the focal cell was detached from the PDMS surface (Figure 2(d)). Cellular detachment was observed on the optical microscope and the images of the cell were recorded through a CCD camera connected to a computer. The cell radii and spreading area were measured by image analysis using the ImageJ software (National Institutes of Health, USA). The electrical potential at which cell detachment occurred was recorded and subsequently converted to the cell adhesion force by a previously published procedure using Equations (1)–(4) [22]. Thirty cells were individually tested at each time point in triplicate.

### 2.5. Immunoprecipitation

For immunoprecipitation from the membrane fraction, the cells were washed twice with ice-cold PBS containing 0.1 μM sodium orthovanadate and resuspended in lysis buffer (10 mM Tris-HCl/150 mM NaCl/1 mM EDTA). The samples were centrifuged for 15 min at 80,000 g. The pellets were resuspended in lysis buffer with 1% Nonidet P-40 and sonicated for 30 seconds. After centrifugation for 90 min, the supernatants (1 mg of protein per mL) were incubated with anti-FAK antibody for 2 h at 4 °C with gentle shaking. The immune complex was then incubated with protein A/G agarose for 1 h and then collected by centrifugation. The agarose-bound immunoprecipitates were washed and incubated in boiling sample buffer containing 62 mM Tris-HCl (pH 6.7), 1.25% w/v sodium dodecyl sulfate (SDS), 10% v/v glycerol, 3.75% v/v mercaptoethanol, and 0.05% w/v bromophenol blue. The samples were then subjected to Western immunoblotting by a previously published procedure [23].

### 2.6. Western Immunoblotting

Cells were lysed with a buffer containing 1% w/v NP-40, 0.5% w/v sodium deoxycholate, 0.1% w/v SDS, and a protease inhibitor mixture (PMSF, aprotinin, and sodium orthovanadate). The total cell lysate (50 μg of protein) was separated by SDS-polyacrylamide gel electrophoresis (PAGE) (12% running, 4% stacking) and analyzed by using the designated antibodies and the Western-Light chemiluminescent detection system (Bio-Rad, Hercules, CA, USA), as previously described [24].

### 2.7. Statistical Analysis

The results are expressed as mean ± standard error of the mean (SEM). Student's *t*-test was performed to compare two groups of data, whereas analysis of variance (ANOVA) followed by Scheffe's test were conducted for multiple comparisons. * *p* values < 0.05 were deemed to be significant.

## 3. Results

### 3.1. The Effect of ECM Components (COL1 and FN) on Cell Adhesion Force

Human bladder epithelial cells, ECV304, were used to investigate the influence of FAK on cell adhesion force. The cell detachment voltage was recorded when the cell was completely detached from the membrane surface. When the electric field was supplied on an adhesive cell, the cell morphology was deformed from a spindle figure (cell adhesion stage, Figure 2(c)) to a round shape (after cell detached from membrane surface, Figure 2(d)). In this study, the 3D finite element field modeling software COMSOL 3.4 Multiphysics was used to calculate the electrical field gradient (∇E) and then converted it to cell adhesion force (Table 1).

**Figure 3.**
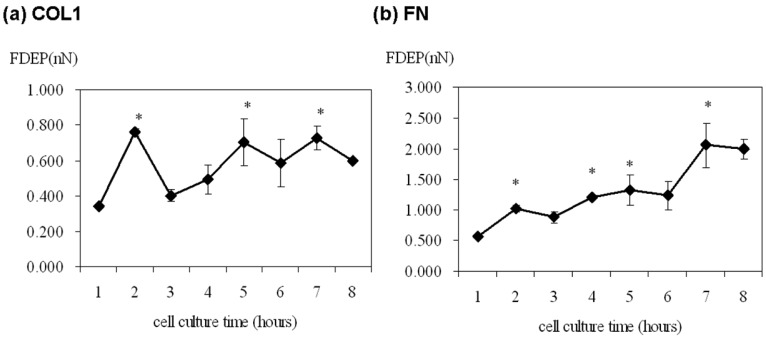
Variation of cell adhesion force (nN) with time of ECV304 cells cultured on (**a**) COL1 and (**b**) FN coated PDMS membranes. Cell detachment force was detected by DEP force measurement system. 30 cells were tested for each cell culture time and each experiment has been repeated three times. * *p* < 0.05 *versus* control (first hour).

The cell adhesion force was significantly high at 2, 5 and 7 h of cultivation on the COL1-coating but was variable over the first eight hours (Figure 3(a)). On the contrary, cell adhesion force on FN-coated membrane gradually increased from 0.577 nN to 2.989 nN with time (Figure 3(b)). Thus, FN showed a more direct effect on ECV304 cell adhesion strength.

### 3.2. The Effect of ECM Components (COL1 and FN) on FAK Phosphorylation

**Figure 4.**
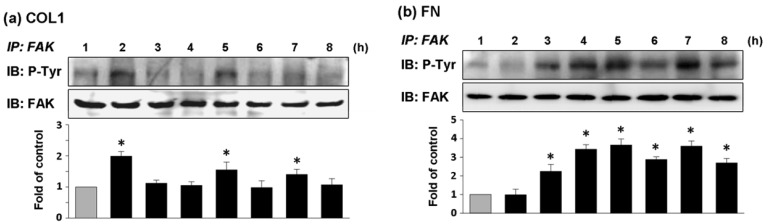
Phosphorylation levels of FAK in ECV304 cells. Cells were cultured on (**a**) COL1 and (**b**) FN coated PDMS membranes as times indicated. Cells were collected from each time point and lysed by lysis buffer and immunoprecipated with FAK antibody as described in Methods and Materials. FAK phosphorylation at first hour was used as control. Phosphorylated FAK level was presented as band densities (normalized to immunoprecipitated FAK protein level) relative to control. Effect of COL1 and FN mediated phosphorylation of FAK was demonstrated. IP, immunoprecipitation; IB: Western immunoblotting. * *p* < 0.05 *versus* control (first hour).

ECV304 cells cultured on COL1-coated membrane had high expression of phosphorylated FAK (pFAK) at 2, 5, and 7 h of cultivation (Figure 4(a)). In comparison, the effect of FN coating on FAK phosphorylation of ECV304 was strongly expressed from the fourth hour of cell cultivation and the expression of pFAK was remained high thereafter (Figure 4(b)). The chronological trends of FAK phosphorylation followed closely with those of cell adhesion force for both types of coated membranes. Thus cell adhesion was closely associated with FAK activation.

### 3.3. The Effect of Tyrosine Kinase Inhibitors on FAK Phosphorylation and Cell Adhesion Force of ECV304 Cells

Two general tyrosine kinase inhibitors, AG18 and genistein were used to investigate the correlation between FAK phosphorylation and cell adhesion force [25,26]. Since the effect of COL1-coating on FAK activation was significant at 2 and 5 h of cultivation, FAK expression inhibition and cell adhesion force reduction were investigated at those times on COL1-coated membranes. Similarly, FAK phosphorylation at 5 and 8 h was investigated in FN-coated membranes. In both cases, FAK expression at the first hour was the control.

**Figure 5.**
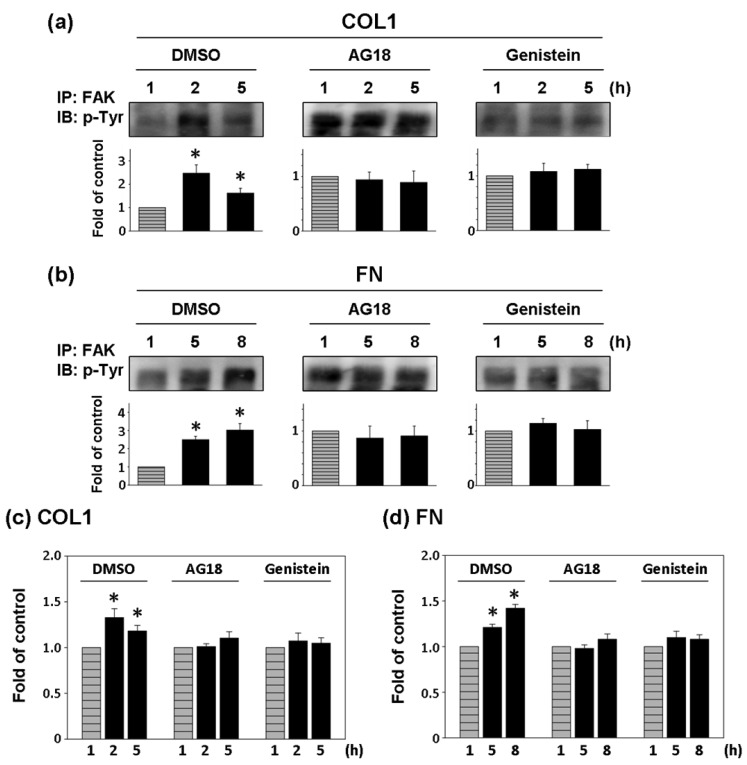
Effect of tyrosine kinase inhibitors, AG18 and Genistein, on COL1 and FN-mediated phosphorylation of FAK and cell adhesion strengthening. FAK phosphorylation and cell adhesion strengthening at first hour cultivation was used as control. (**a**) and (**b**) Cells were pretreated respectively with DMSO, AG18, and Genistein for 1 h and then cultured on (**a**) COL1- or (**b**) FN-coated PDMS membranes in the presence of DMSO or inhibitors as times indicated. Cells were collected from indicated time points and lysed by lysis buffer and immunoprecipated with FAK antibody. Phosphorylated FAK level was presented as band densities (normalized to immunoprecipitated FAK protein level) relative to control. IP, immunoprecipitation; IB: Western immunoblotting. * *p* < 0.05 *versus* control (first hour). (**c**) and (**d**) Cells were pretreated respectively with DMSO, AG18, and Genistein for 1 h and then cultured on (**c**) COL1- or (**d**) FN-coated PDMS membranes in the presence of DMSO or inhibitors as times indicated. Cell detachment force of indicated time points was detected by DEP force measurement system normalized to cell adhesion strengthening at first hour cultivation (which was assigned a value of 1 arbitrary unit). * *p* < 0.05 *versus* control (first hour).

**Figure 6.**
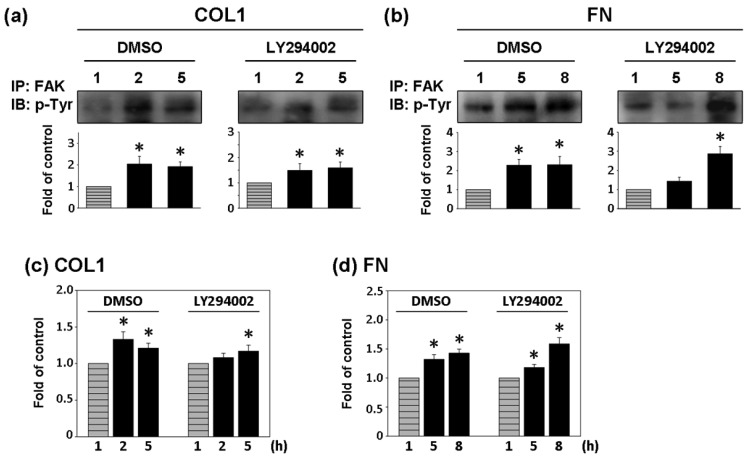
Effect of PI3K inhibitor, LY294002, on COL1 and FN-mediated phosphorylation of FAK and cell adhesion strengthening. FAK phosphorylation and cell adhesion strengthening at first hour cultivation was used as control. (**a**) and (**b**) Cells were pretreated with DMSO or LY294002 for 1 h and then cultured on (**a**) COL1- or (**b**) FN-coated PDMS membranes in the presence of DMSO or LY294002 as times indicated. Cells were collected from indicated time points and lysed by lysis buffer and immunoprecipated with FAK antibody. Phosphorylated FAK level was presented as band densities (normalized to immunoprecipitated FAK protein level) relative to control. IP, immunoprecipitation; IB: Western immunoblotting. * *p* < 0.05 *versus* control (first hour). (**c**) and (**d**) Cells were pretreated with DMSO or LY294002 for 1 h and then cultured on (**c**) COL1- or (**d**) FN-coated PDMS membranes in the presence of DMSO or LY294002 as times indicated. Cell detachment force of indicated time points was detected by DEP force measurement system normalized to cell adhesion strengthening at first hour cultivation (which was assigned a value of 1 arbitrary unit). * *p* < 0.05 *versus* control (first hour).

As shown in Figure 5(a,b), AG18 (100 μM) inhibited the phosphorylation of FAK at 2 and 5 h of cultivation on COL1-coated membranes and at 5 and 8 h of cultivation on FN-coated membranes. Similarly, genistein (60 μM) showed the complete inhibition of FAK phosphorylation in both COL1- or FN-coated membranes. Thus both AG18 and genistein effectively inhibited FAK phosphorylation. Furthermore, Heinz *et al.* reported that the FAK-induced cell movement must be completed by phosphatidylinositol-3-kinase (PI3K; 110 kDa) [27]. Thus, PI3K has an important role in cell migration. Though this study primarily focused on the activation of FAK, PI3K also showed signs of activation at different time points during cultivation. However, the addition of LY294002 (PI3K Inhibitor, 50 μM) only inhibited FAK phosphorylation at the fifth hour of activation in COL1-coated membranes (Figure 6(a)). Moreover, LY294002 had no inhibitory effect on FAK phosphorylation in FN-coated membrane (Figure 6(b)). AG18 showed a significant decrease in cell adhesion force at the second hour of cell cultivation in COL1-coated membranes (Figure 5(c)). On the other hand, cell adhesion force was reduced at the eighth hour of cell cultivation in FN coated membranes (Figure 5(d)). The addition of genistein also decreased cell adhesion force in both COL1- and FN-coated membranes at the times investigated. The reduction of cell adhesion force through tyrosine kinase inhibitors confirmed that cell adhesion was highly correlated with FAK phosphorylation. However, LY294002 (PI3K inhibitor) did not lead to an obvious decrease in cell adhesion force (Figure 6(c,d)). Thus cell adhesion may not be mediated by PI3K.

## 4. Discussion

Cell adhesion is intimately related to cellular characteristics and processes such as morphology, migration, growth, and differentiation. There is increasing evidence that FAK is a key factor in mediating cell adhesion force [6–8]. Michael *et al.* used sheer stress to study the relationship between cell adhesion force and FAK expression. FAK expression in FAK-null cells promoted cell adhesion force by integrin activation [8]. In the present study, DEP force measurement and quantitative biochemical methods were employed in the present study to examine the relationship between FAK activation and cell adhesion force in different ECM coatings. The adhesion force of ECV304 cells in COL1-coated membranes was variable (Figure 3(a) and Table 1). Cell adhesion force was high at 2, 5, and 7 h of cell cultivation, with maximal force achieved at 2 h. On the contrary, cell adhesion force in FN-coated membranes increased with time (Figure 3(b) and Table 1). Cell adhesion force was high at 2, 4, and 7 h of cultivation, reaching maximum at 7 h. In addition, cells cultured in both coating systems showed consistent trends between cell adhesion force and FAK phosphorylation at various time points. Tyrosine kinase inhibitors, AG 18 and genisteine, not only inhibited FAK phosphorylation, but also reduced cell adhesion.

Generally, ECM components such as COL1 and FN bind different types of integrins on the cell surface to influence many aspects of cellular behavior, including differentiation, motility, growth, and survival. Therefore, linkage between integrins and ECM components mediates the adhesion between cells and their environments (e.g., surface materials). Previous studies showed that an increase in cell spreading area was accompanied by higher adhesion force [28]. Thus, the cell radius and spreading area of ECV304 on COL1 and FN coated membranes was recorded each hour by optical microscopy (Table 1). Interestingly, the cell morphologies on COL1- and FN-coated membranes were different but there was no major variation on cell radius or spreading area. In addition, the radii of ECV 304 cells increased from 12–15 μm in the first hour to 12–19 μm in the second hour, with no significant change thereafter. Although cell radii are factors in the calculation of cell adhesion force (Equation (1)), the variation in the radii in the present study may not be significant enough to influence cell adhesion force or FAK phosphylation level. The present study showed that ECV304 cells on FN-coated membranes had stronger cell adhesion (0.577–2.053 nN) than the COL1 counterparts (0.343–0.760 nN) over the first eight hours of cell cultivation. There were higher fluctuations in cell adhesion force for COL1-coated membranes whereas that in FN-coated ones increased with time. Gallant *et al.* and Elineni *et al.* mentioned that cell adhesion force was contributed by focal adhesion size, integrin binding and focal adhesion assembly [29,30]. These findings indicate that different ECM components may vary cell adhesion strength through focal adhesion size, distinct types of integrin binding and focal adhesion assembly for cell spreading, migration and growth.

Cell adhesion force has previously been measured with different methods. Michael *et al.* (2009) measured it by detaching human dermal fibroblasts from PDMS surfaces using a spinning disk and obtained an adhesion force of 10.5 nN (converted from 232 dyn/cm^2^ for a cell radius of 12 μm). Weder also measured it by detaching human osteosarcoma cells, Saos-2 from cell culture dishes using AFM, reporting results of 0.88–1.2 nN [31]. Similar adhesive forces (1–3 nN) between trophoblasts and uterine epithelium were measured by AFM [32]. The cell adhesion forces of ECV304 cells measured in the current study are within the same orders of magnitude to those obtained in the aforementioned studies (Table 1). On the other hand, the DEP force measurement system can record the cell morphology in real-time and measure many samples in a short time more efficiently than the spinning disk and AFM methods. Therefore, DEP force measurement is a viable alternative method to quantify cell-ECM adhesion.

The importance of FAK in regulating cell adhesion force provides new insights on cellular surface-binding models. FAK was identified as a signal transducer mediating the physical properties of the substrate in fibroblasts [33]. In addition, FAK is required for the adhesion of colon cancer cells to liver sinusoids and lung capillaries [34,35]. In the present study, FAK activation was demonstrated to be highly correlated to cell adhesion force. These findings are similar to those reported by Michael *et al.* (2009). When AG18 was added, FAK activation at the second hour of cell cultivation in COL1-coated membranes and the eight hour of cell cultivation in FN-coated ones were inhibited. These observations were mirrored by reductions in cell adhesion force at the corresponding time points. Similarly, genistein inhibited the activation of FAK and cell adhesion in both coating systems. Conversely, LY294002 did not lead to a significant decrease in cell adhesion force. In fact, cell adhesion force increased at certain time points. These results indicated that LY294002 did not affect cell adhesion.

## 5. Conclusions

This study demonstrated the feasibility of using DEP force measurement to investigate cell adhesion force. The data indicated that cell adhesion force of ECV304 cells was highly associated with FAK phosphorylation levels through the addition of inhibitors, AG18 and genistein. Furthermore, the effect of the extracellular matrix (ECM) component on cell adhesion strength and stability can be quantified efficiently by DEP force measurement.

## Figures and Tables

**Table 1.** The cell adhesion force (F_DEP_) of ECV304 cells cultured on COL1 and FN coated PDMS membranes at varied cell culture time, based on the calculated value of $\nabla {\text{E}}_{\text{rms}}^{2}$ in the present study.

**Culture time (h)**	**Radius of cell (μm)**		**Collagen (COL1)**					**Fibronectin (FN)**		

	**major axis**	**minor axis**	**applied potential (V_pp_)**	**calculated ∇ |*E_rms_^2^*| (V^2^m^−3^)**	***F_DEP_* (nN)**	**standard deviation**	**applied potential (V_pp_)**	**calculated ∇|*E_rms_^2^*| (V^2^m^−3^)**	***F_DEP_* (nN)**	**standard deviation**
1	15	12	11.00	8.96 × 10^13^	0.343	0.012	14.27	1.51 × 10^14^	0.577	0.038
2	19	12	14.60	1.57 × 10^14^	0.76	0.021	16.87	2.11 × 10^14^	1.022	0.050
3	19	12	10.60	8.32 × 10^13^	0.403	0.030	15.73	1.83 × 10^14^	0.886	0.093
4	19	12	11.73	1.02 × 10^14^	0.494	0.080	18.33	2.49 × 10^14^	1.206	0.040
5	19	16	12.13	1.09 × 10^14^	0.704	0.135	16.67	2.06 × 10^14^	1.330	0.252
6	19	16	11.10	9.12 × 10^13^	0.589	0.133	16.07	1.91 × 10^14^	1.233	0.232
7	19	16	12.33	1.13 × 10^14^	0.730	0.069	20.73	3.18 × 10^14^	2.053	0.368
8	19	16	11.20	9.29 × 10^13^	0.600	0.000	20.40	3.08 × 10^14^	1.989	0.156
